# Adherence to Epilepsy’s Medical Recommendations

**DOI:** 10.3390/brainsci14030255

**Published:** 2024-03-05

**Authors:** Anna Jopowicz, Agnieszka Piechal, Elżbieta Bronisz, Iwona Kurkowska-Jastrzębska

**Affiliations:** 1 Department of Rehabilitation, Eleonora Reicher National Institute of Geriatrics, Rheumatology and Rehabilitation, Spartańska 1, 02-637 Warsaw, Poland; annajopowicz@gmail.com; 2 Second Department of Neurology, Institute of Psychiatry and Neurology, Sobieskiego 9, 02-957 Warsaw, Poland; broniszelzbieta@gmail.com (E.B.); ikurkowska@ipin.edu.pl (I.K.-J.); 3 Department of Experimental and Clinical Pharmacology, Medical University of Warsaw, Centre for Preclinical Research and Technology CePT, Banacha 1B, 02-097 Warsaw, Poland

**Keywords:** adherence, compliance, epilepsy, treatment

## Abstract

The primary problem in the treatment of epilepsy is poor seizure control. Several studies have shown that non-adherence to doctors’ recommendations regarding drug dosage, time of drug administration as well as lifestyle modifications are the most frequent causes of the persistence or reoccurrence of seizures, other than cases of misdiagnosis and poor drug selection. The aim of this study was to assess the prevalence of non-compliance with medical recommendations, both in relation to medicine dosage, regularity of administration and lifestyle, and also to determine the factors affecting patients with diagnosed epilepsy. This study was carried out on a total of 169 patients diagnosed with epilepsy who were under the care of an outpatient neurology clinic. The assessment of compliance was performed using the Patient Rating of Compliance Scale (PRCS), Clinician Rating Scale (CRS) and authors’ scale. Depending on the scale used, varying degrees of non-compliance were noted. They were as follows—65.3% on the authors’ scale, 10% on the PRCS and 9% on the CRS. The following factors influenced compliance with doctors’ recommendations: type of epilepsy, consumption of alcoholic beverages, frequency of follow-up visits to the neurology clinic, type of pharmacotherapy and number of medicines taken.

## 1. Introduction

Epilepsy is defined as a disorder characterized by a permanent predisposition to the occurrence of recurrent and unpredictable brain dysfunctions, clinically pictured by epileptic seizures [1]. Many problems of patients with epilepsy result from unsatisfactory therapy results [2]. Despite advances in knowledge and the development of new therapies, approximately 30% of patients treated with antiepileptic drugs have persistent recurrent seizures. According to several studies, the most frequent reason for the persistence or recurrence of seizures in patients with epilepsy, other than misdiagnosis and poor drug selection, is failure to follow the doctor’s recommendations regarding either the dosage or intake of medications as well as lifestyle changes [3,4,5,6,7]. Poor adherence to antiepileptic medication is associated with increased mortality, morbidity and healthcare costs. The process of adherence to medical recommendations starts with the patient being registered for a medical appointment and then continues with the purchase of prescribed medications, the administration of medications by the patient according to the prescribed schedule, and in some cases, by lifestyle modification [8,9,10].

Non-compliance may be intentional (based on the patient’s expectations regarding treatment, adverse drug reactions and lifestyle choices) or unintentional when patients fail to follow medical advice due to unawareness, misunderstanding or uncertainty regarding the clinician’s recommendations [11,12,13,14]. The ideal tool for the assessment of compliance with medical recommendations should be affordable, user-friendly, reliable, flexible and practical. However, there is no single measure that meets all these standards due to each of them having specific drawbacks.

Currently, in clinical practice, one of the most frequently used methods for assessing compliance with medical recommendations is questionnaires containing various scales. The scales assessing adherence to recommendations included in the questionnaires are usually verified with other methods of measuring compliance with medical recommendations, both subjective and objective, which allows for a fairly reliable assessment of patients’ compliance with recommendations [15,16]. The reasons for non-compliance with medical recommendations are often multifaceted and caused by several varied factors. They can be categorized into socio-economic factors, those associated with medical staff, disease, treatment, and factors concerning the patient. From a broader perspective, these elements can be grouped into three categories: those concerning the patient, the physician, and the healthcare system [17,18,19,20].

Factors that negatively impact compliance with medical recommendations include lack of understanding and acceptance of the disease and recommendations, lack of involvement in the treatment decision-making process, forgetfulness, stress, concerns about the occurrence of possible adverse reactions, low motivation, insufficient knowledge of medication administration, the lack of sense of a need to treat, adverse effects of drug therapy, perception of health risks associated with the disease, lack of acceptance of follow-up tests, feelings of hopelessness, the fear of addiction, feeling stigmatized due to the illness, smoking or alcohol consumption [11,12,13,15,20,21,22]. The problem of lack of disease acceptance is often addressed in scientific research [23].

A patient’s inclusion in the therapeutic process and their active participation during treatment is an issue that is being discussed more and more often. The participation of the patient in the process of treatment choice is based primarily on the patient’s trust in the doctor and empathy on the part of the doctor. If the doctor treats the patient as an equal partner during therapy selection, the patient is more likely to adhere to recommendations. Thus, improvement in medical recommendation compliance can be achieved thanks to the patient’s motivation and education, as well as the strengthening of trust [24].

The patient’s personal thoughts about the need to take the medicine, the benefits of taking it, and the concerns about possible side effects and costs associated with taking the drug often determine the level of compliance with medical recommendations [25,26,27,28].

Successful treatment improves patients’ quality of life and reduces overall healthcare costs [29].

The aim of our study was to determine the factors that influence adherence to medical recommendations and the frequency of their occurrence in the group of epilepsy patients at the Institute of Psychiatry and Neurology in Warsaw. In our work, we also investigated the relationship between non-compliance of patients diagnosed with epilepsy and their socio-demographic and clinical characteristics, characteristics of pharmacotherapy recommended, smoking and alcohol consumption.

## 2. Material and Methods

This study was carried out from April 2013 to the end of December 2015. A total of 169 patients (90 women and 79 men) took part in this study. This study investigated the extent to which patients take prescribed drugs, both in relation to the dose, dosing breaks and lifestyle. During visits to the Outpatient Clinic, all patients with diagnosed epilepsy and willingness to take part in the study were interviewed on the subject of compliance with pharmacotherapeutic recommendations. An individual questionnaire specifically designed for the purpose of this study to collect basic socio-demographic data, to describe the course of the disease and treatment of the subjects was completed by patients during a visit to the clinic in the presence of the attending physician. The form included questions regarding basic information about the course of the disease, the duration of the disease, the frequency of hospitalization, data on pharmacotherapy, the number of seizures and the number of visits to the Outpatient Clinic. The form was filled in by individual patients twice over a period of 6–18 months. In addition, information on the course of treatment and the medicines used was analyzed based on the available medical documentation.

To assess compliance with medical recommendations, the following scales were used: PRCS, CRS and authors’ scale [30,31].

The PRCS is a five-level scale, according to which the patient themselves assesses to what extent they adhere to medical recommendations. The scale contains the following gradations in descending order: very good, good, sufficient, poor and insufficient. In the questionnaire, the question on the PRCS was:

To what extent did you comply with the doctor’s recommendations regarding the use of medication over the past month?

The CRS is a seven-level scale, in which the attending physician assesses the patient’s compliance with medical recommendations. According to this scale, 1 point means total refusal of treatment while 7 points is full participation. The CRS contained the following grades:

1. Total refusal of treatment
2. Partial refusal of treatment
3. Unfriendly consent
4. Periodic unwillingness
5. Passive consent
6. Moderate participation
7. Full participation

Due to the fact that so far many different scales have been created to assess compliance with medical recommendations in chronic diseases and there is no scale specifically dedicated to epilepsy, the authors of this study using already available questionnaires chose questions that could be used in the assessment of compliance in epileptic patients and composed their own scale. This scale consists of 11 questions regarding compliance with medical recommendations in relation to pharmacotherapy and lifestyle (six direct questions and five indirect questions).

On the authors’ scale, 0 points were awarded for answers indicating compliance with the recommendations and 1 point for other responses (Table 1).

The results were summed up and those who scored 0–3 points on this scale were considered adherent, while those who scored more than 3 points were considered non-adherent (the maximum number of points on the scale was 11; however, in this study, the lowest and highest number of points was 1 and 8, respectively). The cut-off threshold for the authors’ scale was determined based on the ROC curve analysis. The scale had been verified using statistical methods and the determination of the serum drug concentration.

The validation group consisted of 22 patients who were clinically evaluated and the same questionnaire data as with the study group were assessed. The characteristics of the study group are shown in Table 2. Additionally, blood samples were collected in the validation group for the analysis of the serum drug concentration. The distribution of variables regarding socio-economic and demographic factors, clinical presentation, patient-related (including addictions) factors, knowledge of relatives, health care factors and therapy did not differ significantly in the study and validation groups. In addition, the data collected from the study of the validation group was reassessed and the concentration of the drug was determined 10–12 weeks later. The socio-economic and demographic data of the patients participating in this study are presented in Table 2.

The statistical analysis of the obtained results was carried out using the Statistica computer software (v. 10 PL) and MedCalc 15.8. The boundary of the inference error was set at 5% and the results including values below 0.05 were considered statistically significant. The quantitative variables were analyzed using descriptive statistics: clustering measures (median) and measures of dispersion (i.e., interquartile range—IQR, minimum and maximum value, 95% confidence interval—95% CI). The chi square test (χ^2^) was used to assess the relationship between selected (appropriately categorized) factors. In turn, based on the odds ratio calculation, the risk of non-compliance with medical recommendations (assessed by a number of PRCS, CRS, author scales) was determined in the context of the presence of specific factors: socio-economic demographic, patient-related, related to the course of the disease, related to the healthcare system, related to therapy, related to addictions. The Mann–Whitney U test compared the distribution of values (for the independent continuous variables) in individual subgroups of patients (observing and failing to comply with medical recommendations). Using the Spearman’s rank correlation coefficient, the dependencies between selected factors were evaluated.

## 3. Results

A total of 169 patients (90 women and 79 men) took part in this study. This study included adults who had been treated for epilepsy over a period of at least half a year before the study, who were mentally capable of giving informed consent to participate in the study and to reliably complete the questionnaires. In the study group, the median duration of the disease was 14 years (range: 1–62 years). Similarly, the median duration of the disease treatment was 14 years (range: 1–62 years). The most used drug among patients of the Outpatient Clinic was valproic acid (VPA), which was used by 23.08% of patients. Carbamazepine (CBZ) was used by 14.79% of patients. Another drug used was lamotrigine (LTG), which was taken by 8.88% of patients. The combination of VPA and LTG was accepted by 6.51% of people in the study group. 

During the study period, the patients had generalized tonic–clonic seizures (TCSs), focal seizures (FSs), focal dyscognitive seizures (FDSs) and focal secondary generalized tonic–clonic seizures (TCSs). In the study group, 42.6% of patients had primary or secondary generalized seizures. Moreover, 33.1% of patients had TCSs with FDSs or FSs. Also, 17.8% of patients suffered from FDSs or FSs. There were statistically significant differences in the occurrence of particular types of seizures depending on the sex of the examined patients (*p* = 0.0337).

FSs, in comparison to TCSs or FDSs, were significantly more common in women than in men (81.5% vs. 18.5% *p* = 0.0061). The coexistence of generalized seizures secondary or primary TCSs and FSs and FDSs seizures was more frequently observed in younger patients (median age 29 vs. 39, *p* = 0.135) compared to other types of seizures.

In the study group, idiopathic epilepsy was diagnosed in 18% of patients, symptomatic in 58% of patients, and of unknown etiology in 24% of patients.

The group of patients with idiopathic epilepsy was younger than the group with symptomatic epilepsy (median: 30 vs. 40 years, *p* = 0.0017). On the other hand, the diagnosis of symptomatic epilepsy was significantly more frequent in the elderly (medians: 44 vs. 34 years, *p* = 0.0005). In the case of the other assessed variables, no significant link was found with the gender or age of the examined persons.

In more than 60% of patients, the treatment regimen was polytherapy based on two or more antiepileptic drugs. There were statistically significant differences in the number of drugs used, depending on the sex (*p* = 0.0033). Monotherapy was used more frequently in men (61.9% vs. 38.1%) and polytherapy in women (64% vs. 36%). Moreover, 31.95% of patients were on double drug therapy and 27.22% of patients were on triple drug therapy.

The most used drugs were VPA and CBZ. CBZ was significantly more often used by older people (46 vs. 37, *p* = 0.0370, respectively). In most patients (62.13%), there was no change in the course of treatment over the last year. In the remaining patients, most often it was a single change in therapy over the last year (83.02%). Drug resistance was diagnosed in 39.6% of patients, using the definition of the ILAE (International League Against Epilepsy) (drug-resistant epilepsy may be defined as the failure of adequate trials of two tolerated and appropriately chosen and used AED schedules—whether as monotherapies or in combination—to achieve sustained seizure freedom) [7,32]. 

In most patients (70.41%), adverse reactions were reported within the last year. In about half of the patients, only one type of adverse reaction (53.19%) was noted. However, in the remaining 46.81% of patients, there were at least two different side effects. Side effects were significantly more common in people over 40 years of age (respectively, 40 vs. 33, *p* = 0.0074).

Most patients declared having good knowledge of the course of the illness and treatment. A total of 135 patients (around 80%) maintained that they knew what to do if a dose was missed. Similarly, 146 subjects (86.39%) claimed that they had a specific way of taking the drug to avoid taking the medicine incorrectly. Almost 3/4 (73.96%) of respondents answered in the affirmative to the question regarding awareness of situations that increase the frequency of attacks or provoke attacks. Moreover, 2/3 patients (66.27%) declared that they try to avoid such situations. Almost all subjects (96.45%) admitted that they were aware of the consequences that are associated with irregular medication use (Table 3).

Almost 5% of respondents admitted to frequent or very frequent consumption of alcohol. By contrast, 32% of respondents declared that they consume alcohol very rarely and 19% rarely, while 74 patients (43.79%) declared that they were abstinent. However, only 15.3% of patients admitted to the current use of tobacco. The average number of pack-years amongst the smokers was 15.69, with the lowest and highest number of packets been 3 and 44, respectively.

**Figure 1:**
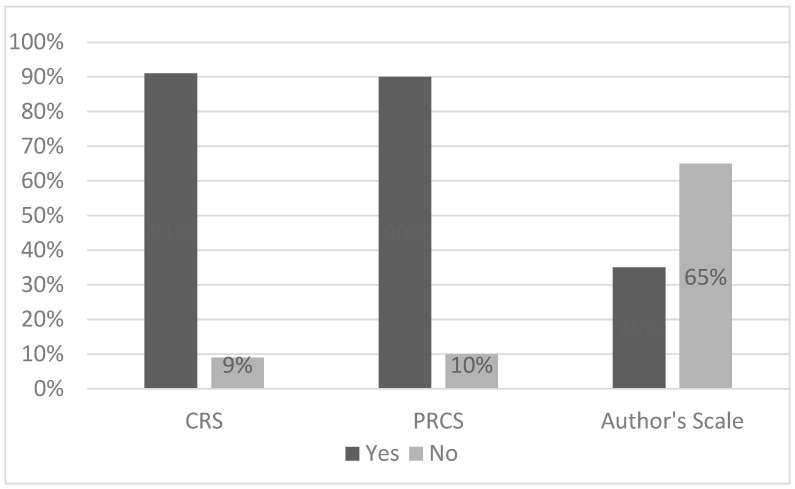
The degree of compliance with medical recommendations assessed according to the individual scales.

Depending on the scale used, the degree of compliance with medical recommendations ranged from 35% to 91% (35% authors’ scale, 90% PRCS, 91% CRS) (Figure 1).

During the PRCS assessment, the overwhelming percentage of patients (slightly above 90%) chose the “very good” response. In turn, the answer “I did not take medicine at all” was given by 6.8% of patients. The answers given by the respondents to the question “To what extent have you observed in the last month the doctor’s recommendations regarding the use of drugs?” are included in Table 4.

In the studied group of patients, of all the analyzed socio-economic and demographic factors, only the source of income significantly influenced compliance with medical recommendations when assessed according to the PRCS. The assessment of patients’ personal circumstances showed that in people whose main source of income was current employment, the risk of non-compliance with medical recommendations was about 3.5 times higher than in patients who lived off their retirement fund, disability pension, social assistance or other sources of income. The other analyzed socio-economic and demographic factors did not significantly affect compliance with medical recommendations when assessed according to the PRCS.

Among the factors related to the course of the disease (the time from the diagnosis of the disease to beginning treatment, the type of epilepsy, the type of seizures, number of hospitalizations due to epilepsy in the last year or the occurrence of drug-resistant epilepsy), only the occurrence of drug resistance significantly affected compliance with the recommendations when assessed with the PRCS. In patients with resistance to treatment, there was almost a 5-fold lower risk of non-compliance with the recommendations of doctors according to the PRCS (OR = 0.22, 95% CI: 0.05–0.99, *p* = 0.0493).

In the case of addiction-related factors, smoking status has proved to have no impact on compliance with medical recommendations when assessed using the PRCS. There was an insignificantly higher (OR = 3.52, 95% CI: 0.96–12.88, *p* = 0.0572) risk of non-compliance with the recommendations among people who declared alcohol consumption (regardless of frequency).

According to the physician’s opinion, the majority of patients (over 47.8%) took full part in the treatment. No patient completely refused treatment; however, 0.6% of patients partially refused treatment. Moreover, 1.8% of respondents gave unwilling consent to treatment. Periodic reluctance toward treatment was reported by 6.9% of patients, while passive consent or moderate participation was recorded for 12.5% and 30.4% of patients, respectively. In the analyzed group of patients, among the evaluated socio-economic and demographic factors, only the age of the patients was significantly related to compliance with medical recommendations/participation in treatment when assessed according to the CRS (the median age of people who complied with recommendations/participation was significantly higher than those who did not follow the recommendations/did not participate in the treatment—39 vs. 33 years, *p* = 0.0211).

Among the factors related to the disease (the time from the diagnosis of the disease to the start of treatment, the type of epilepsy, the type of seizures, number of hospital admissions for epilepsy in the last year or the occurrence of drug-resistant epilepsy), only the type of epilepsy significantly affected the risk of non-compliance with medical recommendations through limited or complete lack of participation in the treatment when assessed according to the CRS. Patients with symptomatic epilepsy had an almost 3-fold lower risk of non-compliance with medical recommendations/limited or total non-treatment when assessed according to the CRS (OR = 0.31, 95% CI: 0.10–0.97, *p* = 0.0439). However, in patients with epilepsy of unknown etiology, there was a more than four times higher risk of non-compliance with medical recommendations/limited or complete non-treatment when assessed according to CRS (OR = 4.39, 95% CI: 1.48–13.03, *p* = 0.0439).

Both among the factors related to the healthcare system as well as those related to therapy, no statistically significant impact on adherence to medical recommendations/level of participation in treatment was assessed according to the CRS. In the case of factors associated with addiction, both the status of smoking tobacco and the consumption of alcoholic beverages proved to have no impact on the compliance with medical recommendations when assessed according to the scale of the CRS.

The level of compliance with medical recommendations assessed on the authors’ scale was 35%. The characteristics of the answers given on the authors’ scale are presented in Table 5.

Almost all the respondents declared the regular purchase of prescribed medication (98.23%). Similarly, nearly 100% of patients responded in the affirmative to the question regarding adherence to the recommended drug dosage. A very high percentage of patients (96.45%) also claimed to undertake additional medical tests as prescribed by the doctor. In addition, the vast majority of patients claimed to attend regular appointments (95.86%).

The answers to the indirect questions cast doubt on the credibility of the answers given to the direct questions. When asked “Do you take medications during an event/trip away from home?”, only 63.9% of respondents answered “very often”. A significant percentage of patients (24.26%) answered “never” to the same question. Also, in regard to the question concerning keeping a seizure diary, almost half of the respondents (48.52%) admitted that they did not. Similarly, only about half of the patients (50.3%) claimed to have a healthy lifestyle. The remaining patients declared that they were trying to lead a healthy lifestyle or had an unhealthy lifestyle (respectively, 41.42% and 5.92% of the respondents).

In the study group, among the analyzed socio-economic and demographic factors, only personal circumstances significantly affected the risk of compliance with medical recommendations when assessed according to the authors’ scale. Analysis showed that in people whose main source of income was their current employment, the risk of non-compliance with medical recommendations was more than 4.5 times higher than in patients who live off other sources of income (OR = 4.58, 95% CI: 2.09–10.03, *p* = 0.0001). However, factors such as sex or living conditions showed only a certain trend toward statistically significant results. Both male sex and living alone were associated with a non-significant (2- and 4.5-fold, respectively) increase in the risk of non-compliance with medical recommendations (respectively, OR = 2.02, 95% CI: 1.00–4.09, *p* = 0.0507, OR = 4.56, 95% CI: 1.00–20.80, *p* = 0.0503). The other analyzed socio-economic and demographic factors did not significantly affect compliance with medical recommendations when assessed according to the authors’ scale.

Four factors relating to the course of the disease (the diagnosis of symptomatic epilepsy, the occurrence of focal seizures without impaired consciousness, drug resistance and the number of hospitalizations due to illness) influenced compliance with recommendations. The incidence of symptomatic epilepsy was associated with an approximately 2.5-fold lower risk of non-compliance with the recommendations compared to the other types (OR 0.42, 95% CI: 0.20–0.89, *p* = 0.0241). However, in patients with focal seizures without impaired consciousness, there was a nearly three-fold reduction in the risk of non-compliance with the medical recommendations compared to patients with other types of seizures (OR = 0.34, 95% CI: 0.14–0.82, *p* = 0.0167). In the case of drug-resistant epilepsy, the risk of non-compliance with medical recommendations was about three times lower than in patients who did not have such resistance (OR = 0.34, 95% CI: 0.16–0.69, *p* = 0.0032). Patients who followed medical recommendations had a nearly three times lower risk of hospitalization (OR = 0.27, 95% CI: 0.07–0.96, *p* = 0.0439).

In the case of the authors’ scale, among the factors related to the patient, avoidance of situations that could cause or exacerbate epileptic seizures significantly modulated the risk of non-compliance with medical recommendations. The risk of failing to comply with medical recommendations was more than 13 times higher in people who failed in avoiding the above-mentioned situations (OR = 13.21, 95% CI: 5.00–34.94, *p* < 0.0001).

In the case of factors related to the healthcare system, the frequency of checkups at the Epilepsy Clinic of the Institute of Psychiatry and Neurology in Warsaw and the physician prescribing drugs had a significant impact on adherence to medical recommendations when assessed according to the authors’ scale. It turned out that the frequent follow-up visits to the epilepsy clinic (at least once in 3 months) reduced the risk of non-compliance about three times (OR = 0.30, 95% CI: 0.11–0.84, *p* = 0.0214). Similar results were obtained if epilepsy drugs were always prescribed by the same doctor, leading to a risk reduction of over five times (OR = 0.19, 95% CI: 0.05–0.67, *p* = 0.0096).

In turn, among the factors associated with the therapy, a statistically significant influence of only one factor, which was the treatment regimen, was observed. The use of polytherapy (two- or three-drug therapy) was associated with a nearly 2.5-fold reduction in the risk of non-compliance compared to patients with only one drug (OR = 0.37, 95% CI: 0.18–0.76, *p* = 0.0065). None of the drugs used influenced the recommendations according to the authors’ scale. However, in situations involving a switch in therapy once in the last year, a statistically insignificant (but showing a trend toward significance) higher risk of non-compliance with the medical recommendations compared to people whose drug regimens were changed more often (two or three times) in the last year (OR = 4.09, 95% CI: 0.83–20.14, *p* = 0.0832) was noted.

In the case of the factors associated with an addiction, smoking status proved to have no impact on the compliance with medical recommendations when assessed according to the authors’ scale. Consumption of alcoholic beverages showed a statistically significant effect on adherence to recommendations. There was a significantly higher (4-fold) (OR = 4.02, 95% CI: 1.92–8.42, *p* = 0.0002) risk of non-compliance by people who declared alcohol consumption (regardless of frequency).

### Validation of the Authors’ Scale

Internal conformity of the tool estimated using the Cronbach’s alpha coefficient.

Based on data on the responses given to individual questions of the authors’ questionnaire, internal consistency was assessed by calculating the Cronbach’s alpha coefficient with the correction of the inverse dependence scales (α = 0.6019, lower limit of 95% CI: 0.3624). The α ≥ 0.5 coefficient indicates the satisfactory compatibility of the internal components of the authors’ scale [16]. The internal compatibility of components of the authors’ scale calculated using the Cronbach’s alpha coefficient is presented in Table 6.

Using the interclass correlation coefficient (ICC), the reliability (accuracy, reliability) of the examined tool was assessed (the results obtained using the authors’ scale were compared in the study group), and then the questionnaire and the serum concentration in all the patients were reassessed after 10–12 weeks (Table 6, question 2). It is assumed that ICC values > 0.60 mean a sufficient level of reliability of the tool evaluated [17]. The values obtained: mean ICC = 0.7920, 95% CI: 0.7105–0.8505 (for single measurements: ICC = 0.6556, 95% CI: 0.5510–0.7398), which indicates the satisfactory level of reliability of the tested tool.

Based on the results of the subjective study (comparison of the results of the survey responses in the test and validation group) using the CCC (Concordance Correlation Coefficient) (calculated on the basis of Pearson’s correlation test), the conformity (described by the precision and accuracy) of the results obtained with of the tested tool was estimated—authors’ scale [18]. The coefficient evaluation showed the near-moderate repeatability of the results (CCC = 0.8779, 95% CI: 0.7520–0.9420). However, the precision and accuracy of the obtained results were high and were, respectively, Pearson’s Pc = 0.9115 and Cb = 0.9632.

Based on the results of the objective study (comparison of the results of the questionnaire responses in the study group and the assessment of the drug serum concentration -tested before its administration, 2 h after administration and 3 months after admission in the validation group), evaluation of the level of agreement of the assessments was carried out (inter-rater reliability, kappa weighted coefficient) [19]. As a result of an objective study, the value of κ = 0.701 (95% CI: 0.541–0.861) was obtained, which indicates the good level of agreement of the assessments.

**Figure 2:**
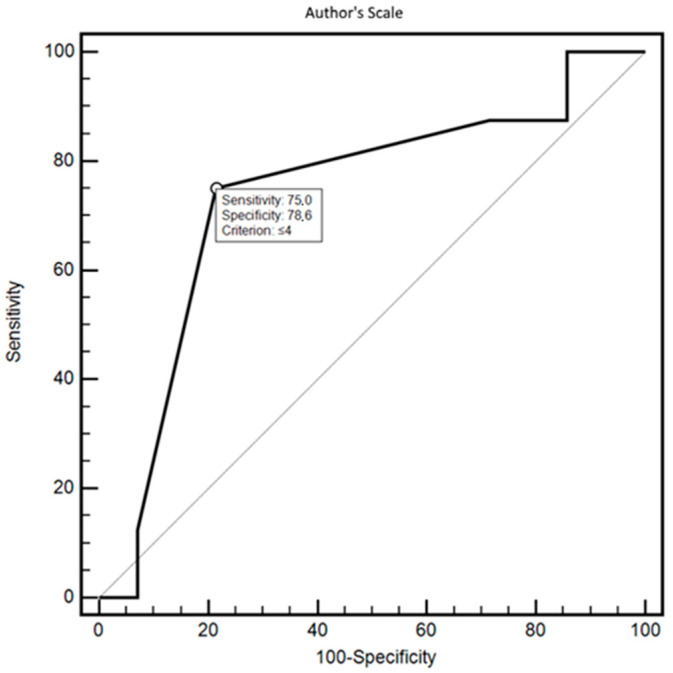
ROC curve of patient differentiation in terms of compliance with medical recommendations based on the authors’ scale (criterion—drug concentration in serum).

The authors’ scale possessed high sensitivity (75%) and specificity (78.6%) in differentiating patients based on compliance with medical recommendations (relative to the rigid criterion determined by the serum concentration of the drug, the criterion for differentiation based on the ROC curve: ≤4 on a scale from 0 to 14), as evidenced by the high AUC value = 0.737 (*p* < 0.0465). The ROC curve for the authors’ scale is presented in Figure 2.

## 4. Discussion

The level of compliance with medical recommendations varied depending on the scale used. It was higher according to the subjective scales (PRCS 90%, CRS 91%) and lower when assessed using an objective scale (authors’ scale) at 35%. The discrepancy between the PRCS and CRS and the authors’ scale can be explained by the fact that the PRCS and CRS contain only one subjective question, based on which the patient evaluates themselves or is assessed by the attending physician. On the other hand, the authors’ scale contains a larger number of questions, considering different aspects of the application of medical recommendations, which allows for a more objective assessment.

In field research conducted in Poland in the years 1969–1974, based on a questionnaire among patients diagnosed with epilepsy, it was shown that 1/3 of patients had seizures, but these patients were never diagnosed nor treated, 1/3 of patients were correctly diagnosed with epilepsy and were treated for this reason, and 1/3, despite the diagnosed epilepsy, stopped treatment arbitrarily. The study also showed that 3–5 people in 1000, despite the occurrence of epileptic seizures, did not take medication, because recurrent seizures did not interfere with daily functioning [33,34].

A study conducted in Ethiopia on a group of over 200 people showed that only 32% of patients adhered to the given medical recommendations, while forgetfulness was listed as the most common reason for missed doses by 75.4% patients [35]. In a study conducted by Das et al., 71% of patients were not adherent to antiepileptic treatment [26]. In a recently published study in Brazilian patients with epilepsy, forgetfulness was also a major factor responsible for non-adherence. Another important factor was the adverse effects of the anti-epileptic drugs taken [28,36,37]. To minimize the risk of skipping a dose, it is recommended to use pill organizers, taking medication during meals and using reminder mobile applications [12,38,39,40,41]. 

In current works, the socio-economic and demographic factors that most often affect compliance with medical recommendations include age, gender, personal circumstances, education and addictions [42,43,44,45]. Among the examined group of people in this study, age and sex had no significant effect on compliance with medical recommendations. According to WHO research, low social status, low income, and illiteracy increase the risk of non-compliance with medical recommendations [46]. In previous studies, data on the influence of the source of income and employment on compliance with medical recommendations were gathered differently [47,48]. Research conducted by Smithson et al. showed that educated and/or people in current employment are less likely to follow medical recommendations [49]. Teh et al. (2020) also found that being employed or a student increased the risk of non-compliance [19,48]. The authors also showed that access to pharmacy services is responsible for non-adherence. In a study conducted in Brazil, among 385 patients with epilepsy, women adhered to medical recommendations better, while men treated their jobs as a priority, which according to the authors of the study resulted in worse compliance with medical recommendations.

In this study conducted among the patients of the epilepsy clinic, a socio-economic and demographic factor that was relevant to compliance with medical recommendations was the source of income. Professionally active patients had a higher risk of non-compliance with medical recommendations: the PRCS showed it as 3.5 times higher, while for the authors’ scale, it was 4.5 times higher. Work, regardless of its nature, often means an active lifestyle in which patients may not have enough time to regularly take medicine and follow medical prescriptions. Patients may have difficulty leaving work to take the medicine at specific times. Additionally, stigma and lack of knowledge of the illness among co-workers may prevent or hinder the regular administration of drugs during working hours. The omission of doses of drugs among working people is also often due to adverse effects of therapy, e.g., drowsiness, which may impede proper job performance. In addition, some patients worked shifts, which contributed to them having irregular hours and in effect increased the likelihood of drug dose omission [48]. 

People who consumed alcohol, regardless of the consumption frequency, had a 4-fold higher risk of non-compliance with the doctor’s recommendations on the authors’ scale. This study showed that patients who consume even small amounts of alcohol have worse compliance with medical recommendations in comparison with abstainers. This situation may be caused by forgetting or deliberately skipping the dose for fear of side effects from combining the drug with alcohol. In several published works on the impact of addictions on compliance with medical recommendations, the negative impact of both smoking and alcohol consumption has been noted [50,51]. A study in Finland showed that smoking and alcohol consumption increased the risk of non-compliance [52]. This is one of the few published studies so far that considers the influence of addictions on compliance with medical recommendations among epileptic patients. Similar results regarding the effect of smoking and alcohol consumption on adherence were also obtained by Duraisamy in a study conducted in 2014 in India. This study showed the negative impact of alcohol consumption on compliance with medical recommendations among patients with tuberculosis and, as in our study conducted in the outpatient clinic, smoking did not significantly affect the compliance with medical recommendations [53]. Sheinfil et al. showed an association between low adherence and alcohol consumption in patients with antiretroviral therapy [51].

The diagnosis of symptomatic epilepsy was a factor that positively influenced compliance with medical recommendations. According to the authors’ scale, symptomatic epilepsy was associated with an approximately 2.5-fold lower risk of non-compliance with the recommendations compared to the others. In turn, the diagnosis of epilepsy of unknown etiology resulted in more than a four times higher risk of non-compliance with medical recommendations. Other results were obtained by Ferrari et al. in a study conducted in a group of 385 people. A total of 316 patients suffered from symptomatic epilepsy and 96 (31.4%) of them complied with the doctor’s recommendations. However, the highest percentage (47.1%) of patients who followed the doctor’s instructions suffered from cryptogenic epilepsy and the smaller number of patients with symptomatic epilepsy (31.4%) complied with the doctor’s recommendations. However, at the same time, patients with symptomatic epilepsy were the largest group among those surveyed by Ferrari et al. (216 people) [47].

Recent studies have shown that non-compliance with medical recommendations amongst both adults and children was more widespread in patients with generalized seizures than in those with focal seizures [54,55]. In our study, similarly to a study conducted in 2013 in China, the type of seizure did not significantly affect compliance with medical recommendations [56]. This result could be due to the majority of patients in the study group having TCSs. In our study group, 42.6% of patients had primary or secondary generalized seizures and 33.1% of patients had TCS attacks co-occurring with FDSs or FSs. Moreover, 17.8% of patients suffered from FDS or FS seizures. However, there were statistically significant differences in the occurrence of particular types of seizures, depending on the sex of the examined patients (*p* = 0.0337).

The factors related to the healthcare system that most often affect the compliance with medical recommendations include lack of access to healthcare [57], long waiting times for follow-up visits [58,59,60], difficulties in obtaining a prescription [60] and dissatisfaction with the course of treatment [59].

In a study conducted by Paschal et al., the frequency of visits to the clinic did not affect compliance with medical recommendations [61]. However, the Gopinath study assessing the impact of doctor–patient communication on compliance with medical recommendations showed that a greater number of visits to the clinic positively influences the doctor’s relationship with the patient and contributes to the improvement of compliance with medical recommendations [62]. Among the surveyed patients, the frequency of follow-up visits to the outpatient clinic at the Institute of Psychiatry and Neurology in Warsaw and the physician assigning drugs had a significant effect on adherence to medical recommendations when assessed according to the authors’ scale. Regular visits to the epilepsy clinic at least once every three months resulted in a three-fold reduction in the risk of non-compliance (OR = 0.30, 95% CI: 0.11–0.84, *p* = 0.0214). In a study on compliance with medical recommendations in relation to regular control visits, it was documented that a lack of regular medical control was associated with a worse prognosis, more severe course of the disease, increased number of hospitalizations and mortality [63,64,65]. Similar results were obtained when antiepileptic drugs were issued continuously by the same doctor; then, the risk of non-adherence decreased by over five times (OR = 0.19, 95% CI: 0.05–0.67, *p* = 0.0096). The results obtained are consistent with the study carried out by Gopinath et al. Patients who are under the constant supervision of one doctor are more likely to follow medical advice. Regular visits to the same doctor have a positive impact on the doctor–patient relationship, heighten the patient’s trust and thusly improve the level of compliance [66].

In the current research, ambiguous results were obtained regarding the effect of the number of drugs taken on compliance with medical recommendations. In most of the published papers, monotherapy increased the chances of compliance with medical recommendations [7,67,68,69,70]. Sweilah et al. and Samsonsen et al. showed that the number of drugs used did not affect compliance with medical recommendations for patients with epilepsy [13,54], while in other studies, the use of more than one drug increased the chances of compliance with medical recommendations [71,72,73]. 

In the conducted study, the use of polytherapy was associated with a nearly 2.5-fold reduction in the risk of non-compliance with recommendations when assessed according to the authors’ scale compared to patients on monotherapy (OR = 0.37, 95% CI: 0.18–0.76, *p* = 0.0065). Similar results were described by Chapman et al. in a study carried out in 2015, in which 1125 people took part. It was shown that patients taking only one drug had a higher risk of non-compliance with the medical recommendations in comparison with patients receiving polytherapy [71]. The obtained result can be explained by the fact that patients receiving only one drug have good seizure control, which contributes to forgetting about medication use and minimizing the disease. In the case of patients receiving polytherapy, the use of one drug was ineffective and as such they had a greater motivation comply with medical advice so as to improve their health. Patients with refractory epilepsy and uncontrolled seizures are often more aware of the need for frequent medication. In addition, these patients require the more active involvement of the attending physician (more frequent visits, longer visits, more tests). In our study, in patients with confirmed drug resistance, the risk of non-compliance with the recommendations when assessed according to the PRCS was almost five times lower (OR = 0.22, 95% CI: 0.05–0.99, *p* = 0.0493), while on the authors’ scale, the risk of non-compliance with medical recommendations was approximately three times lower than in patients without such resistance (OR = 0.34, 0.16–0.69, *p* = 0.0032). Although the level of compliance with medical recommendations among patients of the Outpatient Clinic was higher in patients with confirmed drug resistance than in patients with a good response to treatment, it should be considered if drug resistance, especially in those patients who did not follow the recommendations, did not result from the lack of compliance with medical recommendations [74]. 

In a study in Taiwan conducted in over 13,000 patients over a period of one year, 74% of patients on CBZ, 71% on phenytoin (PHT), 67% on VPA, 56% on oxcarbazepine (OXC), 78% on gabapentin (GBP), 75% on topiramate (TPM) and 54% on lamotrigine (LTG) did not comply with medical recommendations within one year of the inclusion in treatment. This study also showed that compared with patients treated with CBZ, a significantly lower risk of non-compliance was found among patients treated with OXC (adjusted hazard ratio [HR], 0.78; 95% CI: 0.74–0.83), VPA (0.88; 0.85–0.92), LTG (0.72; 0.65–0.81) and TPM (0.90; 0.82–0.98), while patients taking PHT had a significantly higher risk of non-compliance [75,76].

## 5. Conclusions

According to the study conducted, the level of compliance with doctors’ recommendations depends on the scale used. Compliance rated using the authors’ scale, which is a scale composed of both direct and indirect questions allowing the assessment of several parameters making up the recommendations, was low and amounted to 35%.

Among the socio-economic and demographic factors examined, only the source of income significantly influenced compliance with medical recommendations on all three scales—a higher risk of non-compliance with medical prescriptions was found in people who were in current employment. The diagnosis of symptomatic epilepsy was associated with a lower risk of non-compliance with the recommendations compared to other types. In turn, the occurrence of epilepsy of unknown etiology resulted in a more than four times higher risk of non-compliance with medical recommendations.

Frequent visits to the epilepsy clinic (every three months), the use of polytherapy (double or triple) and the occurrence of drug resistance reduced the risk of non-compliance with medical prescriptions in the examined group of people.

In the case of addiction-related factors, smoking was not shown to affect compliance. However, consumption of alcoholic beverages (regardless of frequency) was associated with a significantly higher risk of non-compliance with medical recommendations. As research has shown, it is possible to modify certain factors that increase the risk of non-compliance. 

It can be concluded that particular effort should be made to determine the cause of individual cases of epilepsy and a complete prohibition on alcohol consumption should be advised in regard to epilepsy sufferers. Patients who are suspected of being non-compliant with medical recommendations should have more frequent follow-up visits to the clinic and therapy should be more closely monitored.

## Figures and Tables

**Table 1:** Authors’ scale.

Direct Questions	Indirect Questions
Do you always buy medication prescribed by the doctor?	Do you skip doses if you have not had seizures for a long time?
Have you ever forgotten to take the prescribed medicine?	Do you miss the next dose of medicine when you feel unwell?
Do you ever happen not to take your medicines at the recommended hours?	Do you remember about medications when planning an event/trip?
Has the recommended prescribed drug dosage been followed?	Do you provide a seizure diary?
Do you carry out additional tests as directed by your doctor?	Do you follow lifestyle recommendations?
Do you have regular follow-up visits as planned?	

**Table 2:** Socio-economic and demographic data of the patients and characteristics of the validation group.

Socio-Economic, Demographic, Clinical Data	Variables	Test Group	Validation Group	*p*
Sex (%)	Men	79 (46.75)	8 (36.4)	0.4888
	Women	90 (53.25)	14 (63.6)	
Age (years)	Median [IQR] (range)	37.50 [30–54] (18–84)	48.50 ± [33–58] (19–81)	0.1357
Education level (%)	Higher	52 (30.77)	8 (36.4)	0.7092
	Secondary	79 (46.75)	10 (45.4)	
	Vocational	29 (17.16)	4 (18.20)	
	Primary	9 (5.32)	-	
Main source of income (%)	Salary	70 (41.42)	11 (50)	0.3194
	Retiring/pension	22 (13.02)	5 (22.7)	
	Disablement pension	52 (30.77)	4 (18.20)	
	Social assistance	11 (6.51)	2 (9.10)	
	Other	14 (8.28)	-	

**Table 3:** Characteristics of the test group, including factors related to the patient.

Patient-Centered Factors	Variables	Answers Given by Patients N (%)
Do you know what to do if you miss a dose?	Yes	135 (79.88)
	No	29 (17.16)
	No data	5 (2.96)
Do you have a specific way of administering medication?	Yes	146 (86.39)
	No	20 (11.83)
	No data	3 (1.77)
Do you have any insight into situations that may aggravate or provoke seizures?	Yes	125 (73.96)
	No	40 (23.67)
	No data	4 (2.37)
Do you avoid these situations?	Yes	112 (66.27)
	No	40 (23.67)
	No data	17 (10.06)
Are you aware of the possible consequences if you take medication irregularly?	Yes	163 (96.45)
	No	4 (2.37)
	No data	2 (1.18)
Do you inform your doctor/family of therapy side effects?	Never	14 (8.28)
	Very rarely	10 (5.92)
	Rarely	16 (9.47)
	Often	60 (35.50)
	Very often	48 (28.40)
	No data	21 (12.43)

**Table 4:** Characteristics of the responses given to the PRCS.

Characteristics of Responses Provided to the PRCS	Variables	Answers Given by Patients N (%)
In the last month, to what extent did you comply with your doctor’s recommendations regarding the use of medication? (%)	Very good	146 (90.10)
	Good	2 (1.20)
	Poorly	3 (1.90)
	I did not take medicine at all	11 (6.80)

**Table 5:** Characteristics of responses provided to the author’s scale.

Characteristics of Responses Provided to the Authors’ Scale.	Variables	Answers Given by Patients N (%)
Have you ever missed the administration time of medicines?	Never	61 (36.09)
	Very rarely	58 (34.32)
	Rarely	34 (20.12)
	Often	11 (6.51)
	Very often	3 (1.77)
	No data	2 (1.18)
Do you skip doses if you have not had seizures for a long time? (%)	Never	149 (88.17)
	Very rarely	6 (3.55)
	Rarely	7 (4.14)
	Often	2 (1.18)
	Very often	1 (0.59)
	No data	4 (2.37)
Do you skip the next dose of the medicine when you feel unwell? (%)	Never	156 (92.31)
	Very rarely	6 (3.55)
	Rarely	3 (1.77)
	Often	-
	Very often	1 (0.59)
	No data	3 (1.77)
Have you ever forgotten to take the prescribed medicine?	Never	64 (37.87)
	Very rarely (1–2 times a year)	78 (46.15)
	Rarely (once a month)	16 (9.47)
	Often (weekly)	8 (4.73)
	Very often (>2–3 times a week)	1 (0.59)
	No data	2 (1.18)
Do you always buy medicines prescribed by a doctor?	Yes, I always buy prescription drugs	166 (98.23)
	I almost always buy prescription drugs	3 (1.77)
Are the prescribed medications taken at the recommended doses?	Yes	168 (99.41)
	No, I usually took the medicine at a lower dose	1 (0.59)
	No, I usually took the medicine at a higher dose	0 (0.00%)
Do you carry out additional tests as recommended by the doctor?	Yes	163 (96.45)
	No	5 (2.96)
	No data	1 (0.59)
Do you have regular doctor’s appointments as planned?	Yes	162 (95.86)
	No	4 (2.37)
	No data	3 (1.77)
Indirect questions		
Do you take medications when planning an event/trip?	Never	41 (24.26)
	Very rarely	7 (4.14)
	Rarely	3 (1.77)
	Often	4 (2.37)
	Very often	108 (63.90)
	No data	6 (3.55)
Do you provide a seizure diary?	Yes	72 (42.60)
	No	82 (48.52)
	Irregularly	11 (6.51)
	No data	4 (2.37)
Do you have a healthy lifestyle?	Yes	85 (50.30)
	No	10 (5.92)
	I try to	70 (41.42)
	No data	4 (2.37)

**Table 6:** Internal compliance of the components of the authors’ scale calculated using the Cronbach’s alpha coefficient.

Variable	Alfa	Variation
Have you ever forgotten to take the prescribed medicine?	0.5478	−0.0541
Have you ever missed a dose when you haven’t had seizures for a long time?	0.4021	−0.1998
Do you have a healthy lifestyle?	0.5881	−0.0138
Do you have a seizure diary?	0.5696	−0.0324
Do you take prescribed medicaments at the recommended doses?	0.6012	−0.0007
Do you attend regular medical appointments?	0.6070	0.0050
Do you take medications during trips away?	0.6333	0.0313
Do you miss the next dose of drugs when you feel unwell?	0.3934	−0.2085
Do you carry out additional tests as recommended by your doctor?	0.6070	0.005016
Do you usually buy medicines prescribed by the doctor?	0.6074	0.005493

## Data Availability

The data presented in this study are available on request from the corresponding author. The data are not publicly available because they contain sensitive data. Questionnaires are available in paper form at the Institute of Psychiatry and Neurology in Warsaw.

## Limitations

Due to a lack of funding, the authors could not use already validated tools.
